# Novel functional advanced echocardiography for the assessment of myocardial mechanics in children with neurocardiogenic syncope – a blinded prospective speckle tracking head-up tilt-table challenge study

**DOI:** 10.1186/s12872-018-0826-0

**Published:** 2018-05-08

**Authors:** Kai O. Hensel, Markus Roskopf, Francisca Abellan Schneyder, Andreas Heusch

**Affiliations:** 1 HELIOS University Medical Center Wuppertal, Children’s Hospital, Center for Clinical & Translational Research (CCTR), Faculty of Health, Center for Biomedical Education & Research (ZBAF), Witten/Herdecke University, Faculty of Health, Heusnerstr. 40, D-42283 Wuppertal, Germany; 2 University of Cambridge, Addenbrooke’s Hospital, Department of Paediatrics, Cambridge, UK

**Keywords:** Vasovagal syncope, Collapse, Pediatric echocardiography, Functional autonomic response test, Strain rate, LV function

## Abstract

**Background:**

Data on left ventricular (LV) function in patients with neurocardiogenic syncope (NS) is conflicting in adults and lacking in children. The aim of this study was to analyze LV myocardial performance in children with NS at rest and during head-up tilt-table (HUTT) testing.

**Methods:**

This is the first study to combine HUTT and speckle-tracking echocardiography (STE) in children with NS. 43 consecutive normotensive pediatric patients with NS (mean age 13.9 ± 2.6 years, 51% female) and 41 sex- and age-matched healthy controls were included in the study. The study groups consisted of 21 patients with a positive HUTT reaction (HUTT+) and 22 with a negative HUTT reaction (HUTT-). STE was used to analyze peak systolic LV myocardial strain and strain rate.

**Results:**

Conventional echocardiographic parameters were similar in all analyzed groups. When compared to healthy controls, children with NS had depressed levels of circumferential strain rate (*p* = 0.032) and significantly depressed longitudinal strain rate (*p* <  0.001) at rest. Interestingly, during HUTT testing LV global strain and strain rate were similar in both groups. LV strain rate was lowest in HUTT+ followed by HUTT- and control subjects both at rest and during HUTT.

**Conclusions:**

Resting LV longitudinal strain rate is attenuated in children with NS, especially in those with a positive HUTT response. This is further evidence that NS patients feature altered cardiac mechanics rendering them prone to vasovagal perturbations that can ultimately result in collapse.

**Trial registration:**

Witten/Herdecke University ethics committee clinical study number: UWH-73-2014.

## Background

Syncope is a sudden, brief loss of postural tone and consciousness followed by spontaneous recovery [1]. Up to 15% of children experience an episode of syncope to the end of adolescence [2, 3]. While a few underlying etiologies include potentially life-threatening cardiogenic conditions such as cardiac arrhythmia and structural heart disease, most cases of syncope are of benign origin. Vasovagal syncope - also known as situational, reflex or neurocardiogenic syncope (NS) - is the most common cause of pediatric syncope accounting for more than 50% of cases [4–6]. The development of effective therapies for NS is hindered by the vague and yet conflicting knowledge of the underlying pathophysiologic processes. Sympathetic failure/withdrawal is thought to play a role leading to decreased cardiac contractility and peripheral vascular tone [7]. The “ventricular hypothesis” on the other hand is based on an increase in left ventricular (LV) contractility – i.e. due to ventricular underfilling, sympathetic overactivity or hypovolemia. This may result in LV mechanoreceptor activation, the Bezold-Jarisch reflex, which in turn causes systemic hypotension, peripheral vasodilation and severe bradycardia [8]. However, the origin of the precipitating hemodynamic changes and the specific underlying pathomechanisms resulting in vasovagal syncope in children yet remain a subject of considerable speculation.

Advanced echocardiography techniques – including tissue-Doppler echocardiography (TDI) and speckle-tracking echocardiography (STE) – are promising modalities for the real-time quantitative evaluation of myocardial performance without the disadvantages of adverse effects or high costs. Based on TDI studies, cardiac mechanics such as left atrial and LV filling and contractility are postulated to be altered in neurocardiogenic syncope in adults [9, 10]. STE is reproducible tool for the detection of myocardial strain and strain rate [11, 12]. It has been successfully used to detect subclinical myocardial dysfunction in a variety of clinical settings such as arterial hypertension [13], systemic inflammatory disease [14], diabetes mellitus [15] and short-term alterations of glucose metabolism [16]. In 2013, the “ventricular theory” was fundamentally questioned by Goel et al. as they reported a paradoxical decrease of LV strain in adults with neurally mediated syncope and a positive head-up tilt-table (HUTT) exam utilizing STE [17]. However, echocardiographic assessment of cardiac strain was not performed at the same time as tilt-testing and LV strain rate – which, other than strain, is less dependent of cardiac loading conditions and therefore a better parameter to reflect LV contractility [18] - was not measured. This is the first study utilizing speckle-tracking imaging to shine a light on real-time cardiac mechanics in HUTT testing in children with vasovagal syncope.

## Methods

### Study population

In this prospective study, we altogether enrolled 84 children – 43 consecutive normotensive pediatric patients aged 13.9 ± 2.6 years (51% female) with a history of vasovagal syncope and 41 healthy sex- and age-matched controls (13.3 ± 3.1 years, 53% female) without medical problems and with a normal transthoracic echocardiogram. A priori, the study group was further sub-categorized according to their HUTT exam reaction. Inclusion criteria for the study group were a typical history of NS, age < 18 years and a written informed consent signed by the patient and their legal guardian. Moreover, the presence of any acute or chronic condition that might affect the cardiovascular system (e.g. infections, metabolic conditions such as diabetes mellitus, inflammatory disease, renal disease), ECG abnormalities, chronic medication use and the lack of cooperation to participate in this voluntary HUTT study were strict exclusion criteria. 60 patients were excluded from the study due to various reasons: 53 were incompliant regarding the HUTT challenge, 2 patients were obese (BMI > 30), 2 had insufficient image quality at baseline and in 3 patients the continuous ECG signal was corrupted due to technical difficulties which made consecutive speckle tracking post-processing impossible. The study was carried out in accordance with the declaration of Helsinki’s ethical principles for medical research involving human subjects and approved by the Witten/Herdecke University ethics committee (*clinical study number: 73/2014*).

### Conventional echocardiography

The study took place in a calm room with dimmed lights and at normal room temperature. We performed a comprehensive spectral and color flow Doppler echocardiographic study in all included children according to the current guidelines of the American Heart Association [19]. We used an S5–1 Sector Array transducer (Sector 1–5 MHz) and the commercially available ultrasound device iE33 by Phillips Ultrasound Inc., USA. Echocardiographic images were acquired using a standardized protocol. First, the images and cine loops were digitally recorded in the apical 4-, 3- and 2-chamber views, the parasternal long axis view and in two short axis views at the mitral level and at the level of the papillary muscles. Subsequently, the raw data was transferred to a separate offline workstation for later analysis. XCelera Version 3.1.1.422 by Phillips Ultrasound Inc., USA was used for conventional echocardiographic parameter analyses. M-mode images were recorded and analyzed according to the standardized American Society Echocardiography (ASE) protocol [20]. For the assessment of LV diastolic function pw-Doppler and pw-TDI were utilized and E/A-ratio, E/E’-ratio and mitral deceleration time were measured as previously described [21]. Z-scores were used to evaluate all conventional echocardiographic parameters [22].

### Speckle tracking echocardiography

STE was performed using standard cross-sectional 2D grayscale LV B-mode images to measure strain and strain rate as described previously by our group [23]. Briefly, circumferential strain and strain rate were measured in the standard parasternal short-axis at the papillary muscle plane and longitudinal strain and strain rate were measured in standard apical 4-chamber view. Frame rate was adjusted to 60–90 frames/second and three to five consecutive cardiac cycles synchronized to a continuous ECG were recorded. Caution was paid to minimize artifacts during echocardiographic image acquisition. QLAB Version 10 was used on an off-line workstation to postprocess the anonymized digitally stored DICOM data. Importantly, all involved echocardiographic examiners and interpreters were blinded to the study group status of each participant. Throughout, we verified tissue tracking quality frame-by-frame, in real-time and full thickness coverage of the entire myocardial wall from the endocardial to the epicardial contours was readjusted by hand, where necessary.

### Head-up tilt-table challenge

To unmask potential abnormalities in myocardial performance that might remain undiscovered at rest and to study cardiac mechanics during HUTT gravitational challenge, after the resting conventional and STE exam we additionally exposed the study population to a HUTT test and performed STE simultaneously. The HUTT exam was carried out according to the standard Newcastle protocol as previously described [24]. Specifically, patients were fasted for 2–3 h prior to the study and were first reclined in a flat lying position for at least 10 min. Subsequently, the table was tilted to a 70-degree angle in less than 10 s. Patients were kept in that position for a maximum of 30 min. A positive HUTT test (HUTT+) was defined as the development of presyncope with a significant drop in blood pressure (< 70 mmHg systolic) and the production of clinical symptoms such as dizziness, severe nausea or the sudden loss of consciousness. Strict termination criteria were the patient’s wish to end the exam or the occurrence of a syncope or presyncope. Echocardiographic images were repeatedly acquired in the 70-degrees “hanging” position according to the same standardized scheme of consecutive viewing planes used for the above-mentioned baseline STE assessment. To achieve comparable hemodynamic circumstances for all patients, we analyzed the last recorded echocardiographic images prior to the termination of the study. Peripheral blood pressure measurements were obtained at 2-min intervals and a 12-channel ECG was continuously monitored. The reported hemodynamic parameters (i.e. heart rate, blood pressure) reflect the time at which the echocardiographic images were recorded. After the HUTT test, patients were kept in a lying position for at least 10 min or until the completely recovered (e.g. post-syncopal).

### Biostatistical analyses

Demographic data, baseline clinical parameters, hemodynamics and echocardiographic aspects are presented as mean and standard deviation. Analysis of variance (ANOVA) testing was used to compare clinical, hemodynamic and echocardiographic data of the analyzed groups. Strain and strain rate are displayed as box-whisker-plots. Bonferroni correction was applied to exclude multiple testing bias. Hence, *p*-values < 0.0025 constituted statistical significance. Microsoft Excel Version 16.0 for PC and GraphPad Prism Version 6 (GraphPad Software, Inc., La Jolla, CA, USA) were utilized for all statistical tests.

## Results

### Patient characteristics

Baseline clinical characteristics are outlined in Table 1. Children with NS and a positive HUTT exam (HUTT+) were aged 13.4 ± 2.8 years, HUTT- group mean age was 14.4 ± 2.3 years and healthy controls were 13.3 ± 3.1 years old (*p* = 0.318). Height (*p* = 0.375), body weight (*p* = 0.101), body mass index (p = 0.1) and body surface area (*p* = 0.165) were not statistically different between the three groups. All these values were within normal limits as evaluated by Z-scores. HUTT response was positive in 21 of the 43 patients with NS and 2 of the 41 healthy controls (*p* <  0.001). Hemodynamic parameters, e.g. heart rate and arterial blood pressure did not differ between the three groups neither at rest nor at the moment of STE assessment during HUTT testing (0.2 < *p* < 0.964).

**Table 1:** Baseline clinical characteristics and hemodynamics of the study population

		Syncope (HUTT+) (*n* = 21)	Syncope (HUTT-) (*n* = 22)	Control (*n* = 41)	*p*-value (ANOVA)
	Age (years)	13.43 ± 2.84	14.43 ± 2.31	13.30 ± 3.06	0.318
	Height (cm)	160.14 ± 20.49	164.24 ± 17.06	157.65 ± 15.67	0.375
	Weight (kg)	56.29 ± 21.49	60.10 ± 19.28	49.95 ± 15.34	0.101
	Female gender (%)	66.7%	40.9%	53.66%	0.184
	Body surface (m^2^)	1.57 ± 0.39	1.65 ± 0.34	1.48 ± 0.29	0.165
	Body mass index (kg/m^2^)	21.07 ± 4.66	21.65 ± 3.62	19.62 ± 3.2	0.1
	Positive tilt-table test (n)	21	0	2	0.001
Baseline	Heart rate (bpm)	81.18 ± 18.39	82.27 ± 23.38	80.42 ± 13.33	0.964
	BP systolic (mmHg)	113.12 ± 26.42	122.02 ± 52.76	111.29 ± 19.02	0.436
	BP diastolic (mmHg)	76.02 ± 24.96	77.64 ± 35.85	64.02 ± 18.36	0.2
HUTT	Heart rate (bpm)	93.24 ± 26.7	101.41 ± 27.19	96.82 ± 16.03	0.605
	BP systolic (mmHg)	133.16 ± 45.09	133.71 ± 41.63	128.70 ± 13.56	0.866
	BP diastolic (mmHg)	85.94 ± 34.98	87.06 ± 32.33	77.97 ± 18.09	0.580

### Conventional echocardiographic parameters

Standard 2D derived conventional echocardiographic parameters were similar in all groups (Table 2). In detail, M-mode measurements yielded comparable LA/Ao ratio (*p* = 0.480), fractional shortening (*p* = 0.760), LV dimensions (*p* ≥ 0.15) and LV mass (*p* = 0.578). LV stroke volume was lowest in the HUTT+ group (46.7 ± 17.3 vs. 53.5 ± 21.3 vs. 53.8 ± 17.6 ml) when compared to HUTT- and healthy control subjects. However, this difference was not statistically different (*p* = 0.796). Moreover, LV ejection fraction (EF) was 54.3 ± 2.9% in syncope HUTT+ patients, 54.7 ± 6.6% in HUTT- and 56.7 ± 4.4% in healthy controls (*p* = 0.263). Furthermore, diastolic function as reflected by E/A ratio and E/E’ did not differ significantly between the analyzed groups (*p* ≥ 0.352). Mitral deceleration time was longer in the HUTT- group (0.20 ± 0.07 s, *p* = 0.028) but similar in HUTT+ patients with NS and healthy controls (0.17 ± 0.07 vs. 0.16 ± 0.06 s). However, these values were still within normal limits as evaluated by population-based age-specific reference values [25].

**Table 2:** Conventional echocardiographic parameters derived from two-dimensional and Doppler imaging

	Syncope (HUTT+) (*n* = 21)	Syncope (HUTT-) (*n* = 22)	Control (*n* = 41)	*p*-value (ANOVA)
LA/AoR	1.08 ± 0.13	1.15 ± 0.17	1.07 ± 0.15	0.480
Fractional shortening (%)	34.48 ± 9.65	33.65 ± 9.18	34.02 ± 3.14	0.760
Interventricular septal end-diastolic diameter (cm)	0.97 ± 0.36	0.86 ± 0.29	0.83 ± 0.14	0.150
LV end-diastolic diameter (cm)	4.35 ± 1.26	4.24 ± 1.31	4.41 ± 0.76	0.764
LV posterior wall diameter. Diastolic (cm)	1.01 ± 0.32	0.98 ± 0.31	0.9 ± 0.24	0.244
LV mass (g)	149.65 ± 74.99	130.55 ± 63.00	128.41 ± 60.62	0.578
LV end-diastolic volume (ml)	91.08 ± 29.21	93.59 ± 36.73	94.92 ± 30.29	0.937
Ejection fraction (%)	54.25 ± 2.94	54.69 ± 6.59	56.66 ± 4.43	0.263
Stroke volume (ml)	46.76 ± 17.34	53.49 ± 21.29	53.79 ± 17.61	0.796
E-Wave / A-Wave	2.12 ± 0.68	2.16 ± 0.85	1.95 ± 0.65	0.352
Mitral deceleration time (s)	0.17 ± 0.07	0.20 ± 0.07	0.16 ± 0.06	0.028
E/E’ (cm/s)	7.46 ± 1.11	7.56 ± 2.35	7.25 ± 1.84	0.833

### Speckle tracking echocardiography

**Fig. 1:**
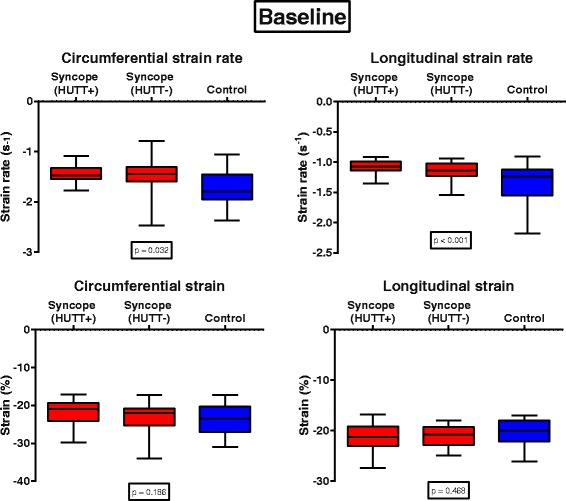
Left ventricular circumferential and longitudinal strain and strain rate at baseline. *p*-values were calculated using the Mann-Whitney-U test

At rest, median peak systolic LV circumferential strain rate (− 1.46 ± 0.47 vs. -1.59 ± 0.69 vs. -1.75 ± 0.47 s^− 1^, *p* = 0.032) and median peak systolic longitudinal strain rate (− 1.08 ± 0.45 vs. -1.16 ± 0.18 vs. -1.39 ± 0.49 s^− 1^, *p* < 0.001) were lowest in children with NS and a positive HUTT exam (HUTT+) as compared to the HUTT- group and healthy controls (Table 3, Fig. 1). In contrast, there was no statistically significant difference between patients with NS and healthy controls regarding peak LV global circumferential strain or global longitudinal strain at baseline.

**Table 3:** Speckle tracking derived peak systolic LV strain rate at rest and during head-up tilt table testing

		Syncope (HUTT+) (*n* = 21)	Syncope (HUTT-) (*n* = 22)	Control (*n* = 41)	*p*-value (ANOVA)
Baseline	Circumferential strain rate (s^−1^)	−1.46 ± 0.47	−1.59 ± 0.69	−1.75 ± 0.47	0.032
	Longitudinal strain rate (s^− 1^)	−1.08 ± 0.45	− 1.16 ± 0.18	− 1.39 ± 0.49	< 0.001
	Circumferential strain (%)	−21.9 ± 7.24	−22.99 ± 7.82	−24.00 ± 5.89	0.186
	Longitudinal strain (%)	−21.31 ± 8.9	−20.94 ± 1.94	−20.48 ± 5.21	0.468
		(*n* = 15)	(*n* = 14)	(*n* = 16)	
HUTT	Circumferential strain rate (s^−1^)	−1.46 ± 0.69	−1.51 ± 0.54	− 1.49 ± 0.25	0.860
	Longitudinal strain rate (s^−1^)	−1.03 ± 0.52	− 1.07 ± 0.28	−1.14 ± 0.59	0.144
	Circumferential strain (%)	−22.24 ± 10.69	−21.98 ± 7.71	− 22.46 ± 3.67	0.915
	Longitudinal strain (%)	−20.48 ± 10.47	−20.86 ± 5.54	− 20.93 ± 10.98	0.926

**Fig. 2:**
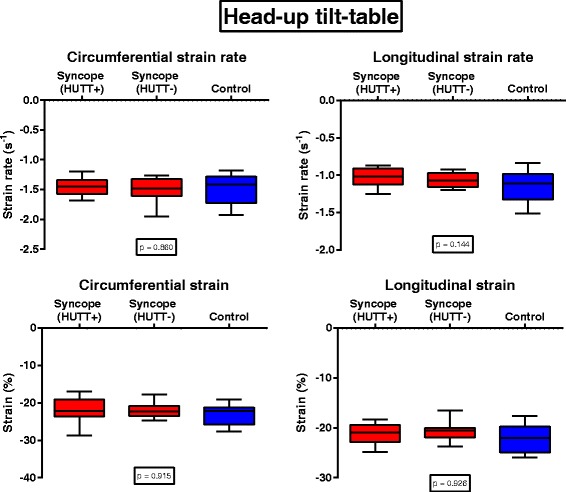
Left ventricular circumferential and longitudinal strain and strain rate during head-up tilt testing. *p*-values were calculated using the Mann-Whitney-U test

**Fig. 3:**
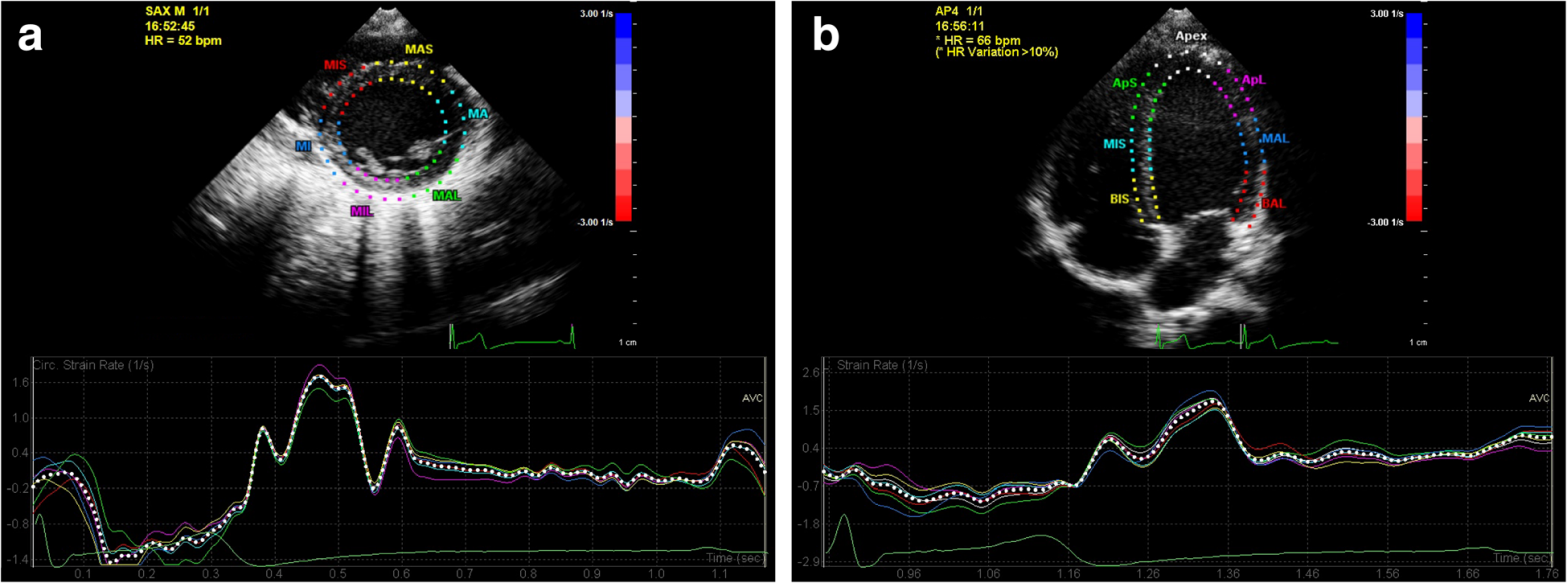
Echocardiographic image examples of speckle tracking derived left ventricular circumferential (**a**) and longitudinal (**b**) strain rate analyses in a patient with neurocardiogenic syncope

During HUTT testing, longitudinal strain rate was lower in children with NS (HUTT+ < HUTT-) than in the control group, but the difference was not statistically different (*p* = 0.144, Fig. 2). Peak LV global circumferential strain and global longitudinal strain were similar in all groups during HUTT testing (Fig. 2). The combination of STE and HUTT was feasible in 15 (72%) HUTT+ patients, 14 (63%) HUTT- patients and 16 (39%) healthy controls. A representative echocardiographic image sample of STE assessment in a child with NS is given in Fig. 3.

While the overall comparison to healthy controls revealed LV myocardial strain rate at rest to be significantly lower in patients with neurocardiogenic syncope, we also observed a heterogeneous distribution of LV deformation in those patients. Interestingly, a subset of patients with NS had high LV circumferential (*n* = 4) or longitudinal strain rate values (*n* = 2; both had also increased circumferential strain rate) at rest (≥ top quartile of healthy controls). Interestingly, all of these patients were in the HUTT- group. One of the four patients was female and one had a history of more than one syncopal episode. The average blood pressure of this subgroup was 121 ± 9 mmHg systolic and 68 ± 2 mmHg diastolic and the average heart rate was 74 ± 10 bpm at. None of the patients with NS and a positive tilt-table response had resting LV longitudinal or circumferential strain rate values ≥ the top quartile of the healthy control group.

## Discussion

This is the first study to simultaneously perform speckle-tracking echocardiography (STE) and head-up tilt-table (HUTT) testing in children with NS. Interestingly, we found LV resting longitudinal strain rate to be significantly lower in NS patients than in healthy controls with a more pronounced decrease in the HUTT+ group. During HUTT challenge we found a similar but not statistically significant tendency of lower longitudinal strain rate in patients with NS. This is in line with findings from Kojo et al., who utilized a Doppler flow meter during HUTT testing and found reduced carotid artery blood flow in pediatric patients with NS [26]. Our findings are furthermore in agreement with results from Sucu and colleagues, who detected atrial conduction delay in adolescents and young adults with NS [27]. Moreover, our data is in accordance with a HUTT echocardiography study in adults reporting that vigorous contraction is probably less responsible for vasovagal syncope release than left ventricle volume reduction [28]. Similarly, HUTT+ NS patients had reduced end-systolic stress, LV volume, and chamber function during HUTT testing in another study, which provides further evidence that if paradoxic activation of LV mechanoreceptors has a role in mediating NS, it is not triggered by increased systolic wall stress or LV hypercontractility [8].

On the other hand, our findings are counterintuitive in the light of the “ventricular theory” in that we did not measure an increase but a decrease in LV contractility in patients with NS and a positive HUTT response. This, however, apart from the fact that the ventricular hypothesis is far from being established conclusively, is in agreement with a similarly performed study by Goel et al., who report attenuated resting LV strain in HUTT+ patients and conclude that – other than previously believed – increased resting LV contractility is not a prerequisite for the development of NS [17]. Notably, even though the authors have measured a similar phenomenon as we did in this study, their study bares significant technical limitations which gives incremental value to our observations. Firstly, Goel et al. have performed STE measurements on a different day then HUTT challenge. Moreover, they utilized LV strain as a measure for LV contractility; strain rate was not assessed. Importantly, STE-derived strain rate has been shown to be more robust to dynamic ventricular unloading than strain in several well-performed animal experimental studies [29–31]. In humans, LV strain has been shown to be affected by changes in cardiac loading conditions such as hemodialysis [32] or gravitational gradients [33]. Furthermore, other than strain, strain rate has been demonstrated to be unaffected by preload and correlate well with end systolic elastance as verified by invasive catheterization [18]. In addition, utilizing a stress testing echocardiography set up, strain rate has been shown to reflect myocardial contractility demonstrating a positive force-frequency relationship in both children and young piglets [34]. In conclusion, our study closes that gap as we performed STE and HUTT on the same occasion and detected LV strain rate in addition to LV strain.

The cardiac autonomic response pattern of patients with NS during HUTT challenge has been distinguished between HUTT+ and HUTT- patients in previous studies [35]. In line with this, we measured more attenuated longitudinal strain rate in HUTT+ patients. The lack of statistical significance for circumferential strain rate and longitudinal strain rate during HUTT is most probably a result of the limited sample size as well as suboptimal image quality during HUTT testing. In the present study, we further found HUTT+ patients to have a lower LV stroke volume when compared to HUTT- and healthy control subjects. This is in accordance with the findings from Moon et al., who described decreased left atrial volumes in HUTT+ patients and concluded a limited intracardiac volume reserve to play an important role in the mechanism of NS [36]. A subset of patients with NS had increased strain rate values – corresponding to a “hyperdynamic” type of NS. Interestingly, none of those patients had a positive response during HUTT challenge. While one could speculate about the pathophysiologic significance of this observation, the small sample size underlying this finding should be taken into consideration. Hence, no definite conclusion can be drawn from this observation at this point. Future studies should further investigate the different types of LV deformation in patients with NS with a specific focus on hemodynamics and cardiomechanics.

Another unresolved issue regarding the enigmatic pathogenesis of NS is the factor age. Even though younger and older patients react differently on the HUTT, especially in the early phase [37], the role of the autonomic nervous system for the etiology of NS seems to be similarly important in adults and children [38]. In extreme cases, the cardioinhibitory effect of the adult autonomous nervous system can reach an extent that can cause death, i.e. cardiac arrest due to extensive inhibitory vagal reflex following a laryngoscopic procedure [39]. Neither has a definite origin in the pathogenesis of neurally mediated syncope been identified, nor do previous studies point toward a definite answer for the “chicken or egg” question. While the “ventricular theory” is centered on the heart as the primarily malfunctioning source, arterial distensibility was also shown to be decreased in patients with recurrent episodes of NS [40]. Similarly, Sucu and colleagues have reported increased aortic stiffness as expressed by altered aortic strain in mid-aged adults with NS and a positive HUTT response [41].

Interestingly, based on the evidence of fear and threat bradycardia in animals and the beneficial vasovagal reflex during hemorrhagic shock in humans and animals, some researchers suggest NS not to be a recently evolved human-only disease but rather the remnant of an ancient defense mechanism that has once reduced myocardial oxygen consumption and supported hemostasis before the development of larger blood losses [42–44]. Under this assumption, it is well imaginable, that this trait is now differentially passed on to subsequent generations and therefore despite being common it is variously pronounced which could explain the different response patterns during HUTT challenge. The principle mechanism during the actual faint is an impaired ability to maintain vasomotor tone in the skeletal muscle blood vessels due to sympathetic withdrawal [45]. While – due to the technical design of this study – we did not perform STE during the event of fainting (HUTT+ group), we assessed LV performance at rest and during HUTT testing before termination of the challenge. The detected lower strain rate may reflect a lower sympathetic tone and thus decreased LV performance which may be a relevant factor in the etiology of NS.

### Limitations

Despite the incremental value of these findings, this study design bares important limitations. Firstly, while STE has been implemented in specific clinical practice of pediatric cardiology, i.e. to monitor cardiotoxicity in chemotherapy patients [46], it is still mostly used in experimental settings. Solid cut-off points for pathologic values, especially for strain rate, are still missing and the technique is subject to relevant inter-vendor inconsistency [47, 48]. Interpretation of STE derived myocardial performance parameter is complex and limited by a variety of factors [49]. Secondly, the combination of STE and HUTT is challenging both for the patient and the involved examiner. When performed according to a standardized protocol, HUTT testing has been shown to be highly reproducible [50]. However, reliable STE measurements require optimal image quality, the absence of artifacts and thorough noise-reduction. We proceeded as previously suggested by limiting deformation analysis to subjects with adequate imaging quality to achieve favorable accuracy [51]. Consequently, as substandard echocardiographic image quality is unavoidable during HUTT testing when the patient is placed in a 70-degree hanging position, not to mention the important factor of patient compliance – especially in children – the results of STE during HUTT challenge must be interpreted with caution. Nevertheless, the most important observation of this study was detected at baseline under normal imaging conditions.

## Conclusion

This study provides further evidence that decreased LV resting performance may be a relevant factor in the etiology of neurocardiogenic syncope in children. Further experimental animal and human studies should be designed to investigate LV contractility in children and adults with NS under different cardiac loading conditions at baseline and in the context of HUTT challenge.

## Abbreviations

ECG: electrocardiogram
EF: ejection fraction
HUTT: head-up tilt-table
HUTT-: pediatric patients with a history of neurocardiogenic syncope and a negative head-up tilt-table response
HUTT+: pediatric patients with a history of neurocardiogenic syncope and a positive head-up tilt-table response
LV: left ventricle
NS: neurocardiogenic syncope
TDI: tissue-Doppler echocardiography
